# Cytokines and Hormones That Contribute to the Positive Association between Fat and Bone

**DOI:** 10.3389/fendo.2014.00070

**Published:** 2014-05-09

**Authors:** Dorit Naot, Jillian Cornish

**Affiliations:** ^1^Department of Medicine, University of Auckland, Auckland, New Zealand

**Keywords:** adipokines, adipose tissue, fat–bone association, pancreatic hormones, gastrointestinal peptides

## Abstract

The positive association between body weight and bone density has been established in numerous laboratory and clinical studies. Apart from the direct effect of soft tissue mass on bone through skeletal loading, a number of cytokines and hormones contribute to the positive association between adipose and bone tissue, acting either locally in sites where cells of the two tissues are adjacent to each other or systemically through the circulation. The current review describes the effects of such local and systemic factors on bone physiology. One class of factors are the adipocyte-secreted peptides (adipokines), which affect bone turnover through a combination of direct effects in bone cells and indirect mechanisms mediated by the central nervous system. Another source of hormones that contribute to the coupling between fat and bone tissue are beta cells of the pancreas. Insulin, amylin, and preptin are co-secreted from pancreatic beta cells in response to increased glucose levels after feeding, and are also found in high circulating levels in obesity. A number of peptide hormones secreted from the gastrointestinal tract in response to feeding affect both fat and bone cells and thus can also act as mediators of the association between the two tissues. The current review focuses on results of laboratory studies investigating possible mechanism involved in the positive association between fat mass and bone mass.

## Introduction

The skeleton provides structural support to soft tissues, attachment sites for muscles, and protection of vital organs. In addition, bone tissue acts as a reservoir for ions that can be sequestered from or released into the serum as required for the maintenance of calcium and phosphate homeostasis. Complex regulatory networks control bone physiology and optimize the response of bone to the changing mechanical and metabolic requirements (1, 2). The size and strength of the skeleton is adjusted to the mass of soft tissue to provide optimal support without being heavier than necessary and the rate of bone turnover has to be constantly adjusted to the availability of nutrients and the levels of circulating calcium and phosphate.

Body weight is positively correlated with bone mineral density (BMD) and negatively correlated with fracture risk (3). In contrast to the consistent finding that underweight is a major risk factor for fracture, the protective effect of increased weight against fracture appears to be more complex. Thus, when analyzing the contribution of the two components of body weight; fat mass and lean mass to the correlation with BMD, most studies identify fat mass as the major contributing factor while some studies find significant correlation with lean mass (4–6). Differential effects of visceral and subcutaneous fat have also been described, suggesting that subcutaneous fat is beneficial to bone whereas visceral fat is deleterious (7, 8). In addition, there is evidence that the relationship between weight and fracture risk is site-specific (9). A recently published study of the relationship of weight, height, and body mass index (BMI) with fracture risk at different sites in post-menopausal women found a significant inverse association between BMI and hip, spine, and wrist fractures and positive association between weight and ankle fractures (10). The effect of body weight on fracture risk also appears to be age-dependent, as obesity is a risk factor for fracture in children but is protective in adults (7).

Obesity is considered a global epidemic with serious co-morbidities that include type-2 diabetes, hypertension, and cancer (11). A number of clinical studies of the relationship between fat and bone mass and fracture risk in obese people demonstrated that the higher BMD in obesity is not as protective against fractures as it is in people with normal BMI (12). During the past two decades, the various procedures of bariatric surgery have increasingly become a common treatment for obesity (11). Both the weight gain in obesity and the rapid weight loss following bariatric surgery have major impacts on the skeleton, and many aspects of the relationship among nutritional status, fat tissue, and the skeleton have been studied in these conditions (11, 13–16).

In recent years, the traditional distinction between endocrine organs and target tissues has become blurred as it is now clear that many tissues secrete factors that circulate and act on target organs, thus creating complex communication networks where many tissues have endocrine functions. In the crosstalk between fat and bone, there is a major role for circulating factors secreted from adipose tissue; adipokines, as well as factors secreted from beta cells of the pancreas and from the gastrointestinal tract in response to feeding. The current review focuses on the bone activities of the local and circulating factors, which generate the positive association between fat and bone.

## Adipokines

Adipokines are regulatory factors secreted from adipocytes that reside within adipose tissue and in the bone marrow. Several adipokines have been studied extensively and their effects in bone and other tissues are well-described, whereas the activities of additional novel adipokines are still largely unknown.

### Leptin

The endocrine function of fat tissue was first discovered with the cloning of leptin and the finding that leptin is secreted from adipocytes and is part of a negative feedback loop that regulates fat tissue mass (17). Circulating levels of leptin positively correlate with body fat and its primary target in the regulation of fat mass was identified as the hypothalamus, where leptin binds to its receptor and regulates appetite and energy expenditure (18). Consequent studies showed a wide distribution of leptin receptors outside the central nervous system, and the expression of the signaling form of the leptin receptor in osteoblasts and chondrocytes suggested the skeleton as a potential target (19–21). The effects of leptin on bone mass are complex, with a combination of direct activity in bone cells, indirect effects through the central nervous systems as well as secondary effects through changes in body mass, and levels of other circulating hormones.

The direct effects of leptin in bone cells are anabolic. Leptin increases proliferation and differentiation of osteoblasts and chondrocytes (19–22) and reduces osteoclastogenesis through inhibiting the expression of receptor activator of nuclear factor κB ligand (RANKL) and induction of osteoprotegerin (19, 23). These results suggest that leptin has an overall positive effect on bone, and could therefore be a possible mediator of the positive correlation between fat and bone tissue.

A number of groups studied the phenotype of leptin-deficient mice (*ob/ob*) and leptin receptor-deficient mice (*db/db*), but the results of these studies are sometimes conflicting, possibly due to the different skeletal sites investigated and the specific parameters measured (1, 3, 24). The two animal models show profound obesity with reduced trabecular bone volume and BMD in femora and tibiae and an increase in the number and size of adipocytes in the marrow (25, 26). Systemic administration of leptin has an overall positive effect on bone; injection into *ob/ob* mice led to an increase in BMD (21, 25), while in wild-type mice leptin injection increased bone strength in males (26) and reduced ovariectomy-induced bone loss in females (27).

In contrast to these studies, Ducy et al. (28) found that circulating leptin inhibits bone formation via the hypothalamus and the sympathetic nervous system. They reported that both *ob/ob* and *db/db* mice have increased bone formation that results in high vertebral bone mass, and that intracerebroventricular (ICV) infusion of leptin causes bone loss in leptin-deficient and in wild-type mice through inhibition of bone formation and stimulation of bone resorption.

The main difference between the studies that show anabolic activity of leptin in bone and the one that demonstrate catabolic effects is the difference in the way leptin was administered: systemically in the former and centrally into the brain in the latter. However, Bartell et al. (29) directly compared the effect of leptin injected SC or through ICV infusion and found that regardless of the way of administration, leptin decreased body weight, food intake, and body fat and increased BMD. Thus, the exact nature of leptin activity in bone remains controversial and requires further investigations (30).

Clinical studies of the effect of leptin in bone are inconclusive, with some finding a positive correlation between leptin serum levels and BMD (31, 32) and others finding no correlation (33–35). Circulating leptin levels were not associated with fracture risk in a large prospective cohort study (36).

### Adiponectin

Adiponectin, produced almost exclusively by adipocytes, is a 28 kDa protein that circulates in humans in trimers, hexamers, and high-molecular weight oligomers, in total concentrations of 0.5–30 μg/mL (37–39). Adiponectin regulates energy homeostasis, glucose and lipid metabolism, and inflammatory pathways (40). Plasma concentrations of adiponectin are inversely related to visceral fat mass and BMI, possibly due to the inhibition of adiponectin secretion by cytokines and hormones that are increased in obesity, by adipose tissue hypoxia, or by a negative effect of adiponectin on its own production (37). Clinical studies consistently show inverse relationships between circulating adiponectin concentrations and BMD, which persist after adjustment for potential confounding factors, including BMI, serum leptin, and central fat mass (33, 36, 41–43). There is a suggestion that an increased level of circulating adiponectin is a risk factor for fracture independent of body composition and BMD (36).

The results of laboratory studies investigating the activity of adiponectin in bone are not entirely congruent, but there is ample evidence that adiponectin affects bone through a combination of direct and indirect mechanisms. The direct activity of adiponectin in bone cells is mediated through the AdipoR1 and AdipoR2 receptors, which are expressed in both osteoblasts and osteoclasts (44, 45). *In vitro*, most groups found that adiponectin stimulates the proliferation and differentiation of osteoblastic cells (46–48), whereas studies of the effect of adiponectin on osteoclastogenesis produced conflicting results. Inhibition of osteoclastogenesis was determined in bone marrow cultures (47, 48) while induction of osteoclastogenesis was found in co-cultures of osteoblasts and CD14 positive cells from peripheral blood (49). Two groups reported a direct inhibitory effect of adiponectin on osteoclast formation in CD14 positive cells and in RAW264.7 cells (47, 50), whereas two studies that used similar experimental systems showed no effects (48, 49). The inconsistency of some of the *in vitro* data could be explained by the finding that commercially available preparations of adiponectin are contaminated with lipopolysaccharide, and that this contaminant is responsible for some of the reported *in vitro* effects observed (51, 52).

A number of animal models were used to investigate adiponectin activity in bone. Adiponectin-deficient mice showed no significant abnormality in bone phenotype at 8 weeks of age (48, 53), but at 14 weeks, micro-computed tomography demonstrated increase in trabecular bone volume and trabecular number (48). Transgenic mice over-expressing adiponectin in the liver showed no bone abnormality (53), whereas in another study transient over-expression of adiponectin in mice increased trabecular bone mass and reduced osteoclast number and bone resorption (50). A recent *in vivo* study showed that the effects of adiponectin in bone are age-dependent (54). In young animals, the dominant effect of adiponectin is the attenuation of bone formation through inhibition of osteoblast proliferation and induction of their apoptosis, whereas in older animals, these local effects are masked by the activity of adiponectin to decrease the sympathetic tone, indirectly leading to an increase in bone mass. Thus, according to this study, adiponectin has both positive and negative effects on bone mass and adiponectin-deficient mice show increase in bone mass at a young age and an osteoporotic phenotype at 36 weeks (54). In another recent study, Wang et al. (55) compared the effect of ovariectomy on BMD and biomechanical strength in wild-type and adiponectin-deficient mice. The study showed that adiponectin-deficiency protects against ovariectomy-induced osteoporosis in mice, and suggested that a possible underlying mechanism for that effect is the enhanced osteogenic differentiation of mesenchymal stem cells identified in the adiponectin-deficient mice.

### Other adipokines

Resistin, fasting-induced adipose factor (FIAF), visfatin, vaspin, and apelin have been identified in recent years as additional factors secreted from adipocytes (56). Only a few clinical studies investigated the relationship between circulating levels of these factors and BMD, and studies of their activities in bone cells *in vitro* are still in preliminary stages. Most of the clinical studies that investigated the relationship between a number of the adipokines, including apelin, resistin, and visfatin and BMD found no significant correlations (34, 35, 57). A systematic review and meta-analysis found no association between resistin or visfatin and BMD and the authors concluded that inconsistent associations between these adipokines and BMD are probably confounded by body composition, in particular fat mass parameters (58).

The activities of FIAF, resistin, and visfatin in bone cells have been studied *in vitro*. FIAF is cleaved *in vivo* into an N-terminal coiled-coil domain [FIAF (CCD)] and a C-terminal fibrinogen-like domain [FIAF (FLD)] (59, 60). FIAF (CCD) inhibited osteoclast differentiation and function in mouse primary bone marrow and in RAW264.7 cell cultures, and decreased expression of genes encoding key osteoclastogenic factors such as M-CSF, connective tissue growth factor (CTGF), NFATc1, and DC-STAMP (61). FIAF (FLD) and intact FIAF were without effect in these experimental systems. In addition, FIAF (CCD) stimulated the proliferation of rat primary osteoblasts, but had no effect on osteoblasts differentiation. Thommesen et al. found that resistin is expressed in primary human bone marrow stem cells and in mature human osteoblasts (62). The expression of resistin mRNA in RAW 264.7 is increased during differentiation into mature osteoclasts, and treatment with recombinant resistin increased the number of differentiated osteoclasts in RAW 264.7 cells and enhanced the proliferation of MC3T3-E1 osteoblast-like cells. Studies of visfatin, an adipokine highly expressed in visceral fat and up-regulated in obesity and type-2 diabetes, showed that it has insulin-mimetic effects in various cell lines. In human primary osteoblasts, similar to insulin, visfatin enhanced glucose uptake, proliferation, and type I collagen synthesis (63). Further studies of the different adipokines and their effects in bone *in vitro* and *in vivo* are required to determine their possible role in the positive association between fat and bone.

## Beta-Pancreatic Hormones

Insulin resistance in obesity leads to hypersecretion of insulin from β-pancreatic cells and two other hormones co-secreted with insulin, amylin, and preptin, also circulate at high levels in obesity. As these three β-pancreatic hormones directly affect bone cells they are likely to contribute to the fat mass/bone mass relationship.

### Insulin

A large number of clinical studied in women and men determined that circulating insulin concentrations are related to BMD (64–68). Osteoblasts express insulin and IGF-1 receptors and *in vitro*, insulin directly stimulates osteoblast proliferation (69, 70). When administered locally over the calvarias of adult male mice, insulin produces two- to three-fold increases in histomorphometric indices of bone formation (71).

In recent years, insulin signaling in bone was the focus of a number of studies that proposed coupling between bone remodeling and glucose and energy metabolism mediated through an endocrine function of bone cells (72–74). In rodent models, insulin induces the expression of osteocalcin in osteoblasts, and inhibits the expression of osteoprotegerin, therefore stimulating osteoclast activity. Osteoclast-mediated resorption releases uncarboxylated osteocalcin into the circulation, where it ultimately enhances pancreatic insulin production (73, 75). Uncarboxylated osteocalcin is therefore a hormone secreted from bone, which acts as a positive regulator of insulin secretion, and was also shown to promote insulin sensitivity in peripheral tissues. However, the relevance of these novel findings to human physiology is uncertain. Antiresorptive therapies for osteoporosis reduce bone formation markers, including osteocalcin, and therefore patients treated with these medications would be expected to have increased risk for insulin resistance and diabetes. However, a *post hoc* analysis of large randomized placebo-controlled trials of antiresorptive therapies found that suppression of bone resorption, which significantly reduces the level of circulating osteocalcin, does not have a clinically important effect on fasting glucose or diabetes incidence (76).

In addition to its direct activity in bone cells, insulin also affects BMD indirectly, through increasing the levels of circulating sex hormones and saturated fatty acids (1). Clinical studies show that women with hyperinsulinemia have increased production estrogen and androgen in the ovary and reduced production of sex hormone binding globulin in the liver. These indirect mechanisms contribute to the high BMD in states of hyperinsulinemia, including obesity and generalized lipodystrophy (1).

### Amylin

Amylin is a peptide hormone that belongs to the calcitonin family and is evolutionary related to insulin. *In vitro*, amylin directly stimulates osteoblast proliferation (77) and inhibits osteoclast differentiation and activity, although with lower potency than calcitonin (78). Systemic administration of amylin substantially increases bone volume in mice (79) and rats (80). In a study of the osteogenic effects of amylin in OVX rats, amylin protected the animals against OVX-induced bone loss at the distal metaphysis and total femora (81). Higher circulating levels of osteocalcin and lower levels of urinary deoxypyridinoline excretion in the amylin-treated animals in comparison to OVX untreated rats, suggests that amylin acts through the stimulation of osteoblast activity as well as inhibition of bone resorption. A recent study tested the osteogenic activity of amylin in normal rats, fructose-induced insulin-resistant and streptozotocin-induced type-2 diabetic rats (82). Infusion of amylin for 3 days had different outcomes in the three groups of animals. In normal rats, amylin induced an increase in bone formation rate and reduced osteoclast surface and erosive surface in the femur, in type-2 diabetic rats amylin normalized trabecular structure parameters and increased osteoblast number, whereas in insulin-resistant rats amylin appeared to have no osteogenic effects (82).

The phenotype of amylin-deficient mice was described by Dacquin et al. (83). In this model, amylin deficiency did not alter the regulation of food intake, body weight, and glucose metabolism. At the age of 24 weeks, amylin-deficient mice had a typical osteoporotic phenotype; showing decreased BMD of long bones, low bone mass, and a 50% decrease in trabecular bone volume. The number of osteoblasts and bone formation rate were similar in amylin-deficient mice and wild-type controls, suggesting that the osteoporotic phenotype was not a result of a defect in bone formation. The amylin-deficient mice had an increased number of osteoclasts and an increase in degradation products of collagen in the urine, suggestive of accelerated bone resorption. Further studies of amylin-deficient mice demonstrated that the bone effects were sex-dependent. Amylin-deficient males showed increased trabecular thickness at 4 and 6 weeks of age and increased femoral length at 26 weeks, whereas female mice were no different than the wild type (84).

Only a small number of studies investigated the bone effects of amylin in humans. In a study of 16 women with anorexia nervosa and 15 healthy controls, Wojcik et al. found that amylin levels were positively associated with BMD and *Z*-scores at the femoral neck and total hip, and the association remained significant after controlling for weight or fat mass (85). In addition, the study found that women with anorexia nervosa had significantly lower levels of fasting amylin, suggesting that amylin could be one of the mechanisms underlying bone loss in anorexia nervosa (85). In another study, a group of patients with type 1 diabetes were treated with pramlintide (amylin analog) for 12 months (86). Although these patients were expected to benefit from the therapy due to the chronic amylin deficiency associated with diabetes, no change in BMD or biochemical markers of bone metabolism was observed following the treatment.

### Preptin

Preptin, a peptide that corresponds to Asp69–Leu102 of pro-insulin-like growth factor-2 (pro-IGF-2), was identified as an additional molecule stored in secretory vesicles of pancreatic beta cells, and co-secreted with insulin and amylin (87). Preptin increases glucose-mediated insulin secretion (87). Circulating levels of preptin were found to be higher in diabetic patients compared to patients with impaired glucose tolerance and controls, suggesting a potential role for preptin in the pathogenesis of type-2 diabetes mellitus (88). Similar to the other beta-pancreatic hormones, preptin has anabolic activity in bone.

*In vitro* studies demonstrated that preptin stimulates the proliferation of primary rat osteoblasts and osteoblast-like cell lines and reduces osteoblast apoptosis induced by serum deprivation (89). Preptin-induced phosphorylation of p42/p44 MAP kinases in osteoblastic cells and its proliferative effects were blocked by MAP kinase inhibitors (89). In human primary osteoblasts, preptin promoted proliferation and alkaline phosphatase activity through induction of p42/p44 MAP kinase and CTGF (90). Preptin did not affect bone resorption in mouse bone marrow cultures (89). In contrast, a recent study that compared the effect of IGF-I, IGF-II, insulin, and preptin on human bone cells, suggested that preptin induces differentiation and activity of both osteoblasts and osteoclasts (91). *In vivo*, local administration of preptin increased bone formation and bone area in adult male mice (89).

Indications for the anabolic effect of preptin on bone in humans arise from a number of clinical observations. Increased circulating levels of a pro-IGF-2-(89-101) peptide in complex with IGF-binding protein-2 have been implicated in osteosclerosis observed in a number of patients with chronic hepatitis C infections (92). Patients with excess of other forms of pro-IGF-2, which do not contain the preptin sequence, do not develop osteosclerosis. A recent study measured preptin levels and bone metabolic markers in serum samples from male patients with osteoporosis, osteopenia, and normal bone mass (93). Serum preptin levels were lowest in the osteoporosis group, and were positively correlated with bone formation markers, whereas no correlation was observed with markers of bone resorption.

## Gastrointestinal Hormones

Diet plays a critical role in mineral homeostasis as bone turnover is acutely responsive to food intake (94, 95). Feeding results in a decrease of up to 50% in markers of bone resorption and a more modest decrease in markers of bone formation (94, 96). Markers of bone resorption decrease 20 min after food intake and return to baseline after 6 h. In a study of controlled food intake over 5 days, a 70% energy restriction produced about 30% decrease in markers of bone formation and a similar increase in markers of bone resorption (97). A number of peptide hormones secreted from the gastrointestinal tract in response to feeding affect bone tissue, either directly or indirectly, and thus contribute to the coupling between feeding status and bone turnover.

### Ghrelin

Ghrelin, a growth hormone (GH) secretagogue, is a peptide hormone primarily synthesized by cells in the stomach and released in response to fasting, such that circulating levels are maximal prior to meals and fall upon feeding (98, 99). *In vivo*, ghrelin is an orexigenic factor, increasing food intake and inducing release of GH from the pituitary via the hypothalamus.

The ghrelin receptor, GHS-R, is expressed in osteoblastic cells, but we found that ghrelin only weakly activated cell proliferation in cultures of primary rat and human osteoblasts (100). Other groups have shown that ghrelin increases proliferation and differentiation in cultures of animal and human osteoblastic cells (101–105). There is also evidence for ghrelin expression and secretion by osteoblast (101). The effect of ghrelin on bone was studied in a number of animal models. Ghrelin infusion increased BMD in rats, and a similar effect was observed in GH-deficient rats, suggesting the existence of a GH-independent effect of ghrelin in bone (102). In contrast, ghrelin knockout mice and GHS-R knockout mice had no changes in either bone mineral content or density in comparison to control mice (106, 107). Transgenic mice over-expressing ghrelin in the brain and stomach showed increased food intake and impaired glucose tolerance but otherwise had normal body size (108). No analysis of bone metabolism was carried out in this study.

In humans, 4 h of ghrelin infusion in healthy subjects and post-gastrostomy patients showed no acute effects on markers of bone turnover (109). Treatment with an oral ghrelin mimetic for 12 months was associated with a small increase in femoral neck BMD, whereas no changes were observed in other sites (110).

### Glucose-dependent insulinotropic peptide

Glucose-dependent insulinotropic polypeptide or gastric inhibitory polypeptide (GIP) is a 42 amino acid protein secreted from K-cells of the duodenum and from the small intestine in response to feeding, and induces insulin and glucagon secretion from the pancreas (111).

GIP receptor is expressed in osteoblasts, osteocytes, and osteoclasts (95) and *in vitro*, GIP increases osteoblast number and activity and inhibits osteoclast activity, suggestive of an overall direct anabolic effect in bone (112–114). Transgenic mice over-expressing GIP have increased bone formation, decreased bone resorption, and increased bone mass, and a long-term study also demonstrated reduced bone loss with aging (115, 116). In addition, administration of GIP reduces bone loss in ovariectomized mice (112). Mice lacking the GIP receptor, who have blunted insulin response to feeding, have low bone mass with reduced bone formation and increased numbers of mature osteoclasts (117, 118). In humans, 48 h after an intravenous bolus of GIP, there were no significant changes in serum levels of either markers of bone resorption or bone formation (119).

### Glucagon-like peptides

Glucagon-like peptide-1 (GLP-1) and GLP-2 are two hormones derived from proglucagon precursor by proteolytic cleavage. These hormones are co-secreted from intestinal L-cells and their levels rise rapidly after food intake. Similar to GIP, GLP-1 stimulates insulin secretion from the pancreas, whereas GLP-2 acts in the intestine to stimulate mucosal growth and nutrient absorption (94, 95). A number of GLP-1 analogs are being developed as pharmaceutical agents for the treatment of type-2 diabetes, as GLP-1 increases postprandial insulin secretion, suppresses postprandial glucagon secretion, and delays gastric emptying (120). A small clinical study compared the postprandial plasma concentrations of GLP-1 and GLP-2 between a group that had previously consumed calcium phosphate-enriched diet for 3 weeks and a control group. The study showed that postprandial GLP-1 and GLP-2 levels were significantly increased in the group that had calcium phosphate-enriched diet, whereas levels of insulin and glucose showed no differences between the groups (121).

Although GLP-1 receptor is expressed in bone marrow stromal cells and in immature osteoblasts, it is not expressed in mature osteoblasts and therefore it is unlikely to have direct activity in these cells (113, 122, 123). Evidence from *in vitro* experiments suggests no direct effect of GLP-1 in either osteoblasts or osteoclasts (124). However, GLP-1 receptors are expressed in thyroid C cells, where GLP-1 induces postprandial calcitonin release and thus indirect inhibition of bone resorption (124). Mice deficient of the GLP-1 receptor have cortical osteopenia and increased osteoclast numbers and bone resorption activity (124).

In contrast to GLP-1, GLP-2 receptors are expressed on osteoclasts and GLP-2 has been shown to decrease bone resorption *in vitro* (125). In several human studies, administration of exogenous GLP-2 reduced bone resorption, as measured by reduction in circulating bone turnover markers whereas bone formation markers were unaffected (119, 126, 127).

## Conclusion

**Figure 1:**
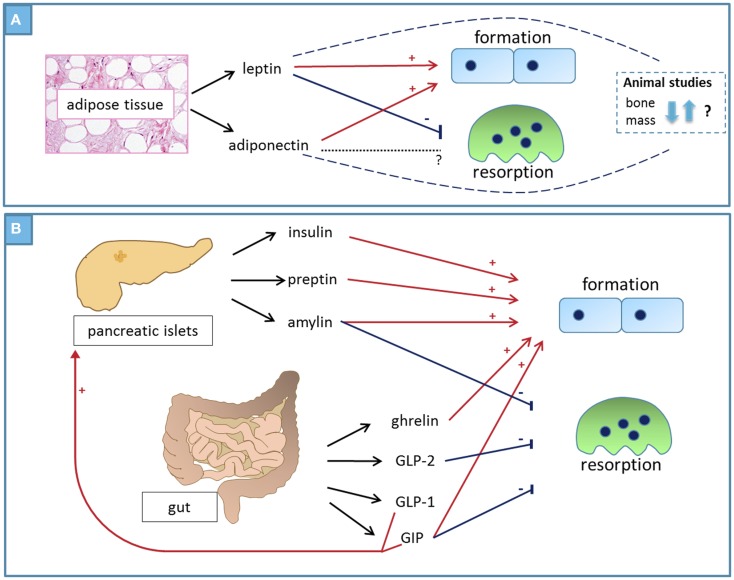
**Summary – factors that mediate the positive association between fat and bone**. **(A)** Adipokines: leptin directly increases osteoblast number and activity while inhibiting osteoclast activity, producing an overall anabolic bone effect. Animal studies produced conflicting results, some suggesting anabolic effect on bones and others showing negative bone effects through indirect mechanisms. Adiponectin – circulating levels of adiponectin correlate negatively with body fat and there is strong inverse relationship between circulating adiponectin and BMD. Adiponectin directly increases osteoblast number and activity, whereas its effects in osteoclasts vary in different experimental systems. The bone phenotype of adiponectin-deficient animals varies with age. **(B)** Peptide hormones secreted from pancreatic islets and from the gut in response to feeding have direct and indirect anabolic bone effects.

The mechanisms of communication between different tissues in the body are very complex and the various *in vitro* and *in vivo* experimental systems designed to investigate these mechanisms do not always produce congruent results. In the study of the relationship between fat mass and bone mass, the most consistent clinical observation is that body weight is positively correlated with BMD and negatively associated with fracture risk. Of the numerous laboratory studies of the possible mediators of the fat–bone association, the current review focused on studies of the direct bone effects of adipokines (summarized in Figure 1A) and of hormones that respond to feeding (Figure 1B). Hormones secreted in response to feeding shift bone turnover balance to favor formation, whereas low levels of these hormones induce bone resorption. It is likely that these responses have evolved to promote bone mass and strength when nutrition is abundant and to maintain calcium homeostasis in times of food shortage. Other important physiological links between fat and bone, which were not discussed in detail here, include their indirect communication through the central nervous system, and the fact that osteoblasts and adipocytes differentiate from a common mesenchymal precursor cell. *In vitro* studies in combination with investigations of genetically modified animals substantially advanced our understanding of the relationship and crosstalk between fat and bone tissue. However, only clinical studies can ultimately determine whether hypotheses produced using experimental model systems have relevance to human physiology and pathology.

## Conflict of Interest Statement

The authors declare that the research was conducted in the absence of any commercial or financial relationships that could be construed as a potential conflict of interest.
